# Barriers and solutions to online learning in medical education – an integrative review

**DOI:** 10.1186/s12909-018-1240-0

**Published:** 2018-06-07

**Authors:** Diane O’Doherty, Marie Dromey, Justan Lougheed, Ailish Hannigan, Jason Last, Deirdre McGrath

**Affiliations:** 1 Graduate Entry Medical School, University of Limerick, Limerick, Ireland; 2 School of Medicine, University College Dublin, Dublin, Ireland; 3 University College Dublin, Dublin, Ireland

**Keywords:** E-learning, Online learning, Medical education, Medical faculty, Barriers, Solutions

## Abstract

**Background:**

The aim of this study is to review the literature on known barriers and solutions that face educators when developing and implementing online learning programs for medical students and postgraduate trainees.

**Methods:**

An integrative review was conducted over a three-month period by an inter-institutional research team. The search included ScienceDirect, Scopus, BioMedical, PubMed, Medline (EBSCO & Ovid), ERIC, LISA, EBSCO, Google Scholar, ProQuest A&I, ProQuest UK & Ireland, UL Institutional Repository (IR), UCDIR and the All Aboard Report. Search terms included online learning, medical educators, development, barriers, solutions and digital literacy. The search was carried out by two reviewers. Titles and abstracts were screened independently and reviewed with inclusion/exclusion criteria. A consensus was drawn on which articles were included. Data appraisal was performed using the Critical Appraisal Skills Programme (CASP) Qualitative Research Checklist and NHMRC Appraisal Evidence Matrix. Data extraction was completed using the Cochrane Data Extraction Form and a modified extraction tool.

**Results:**

Of the 3101 abstracts identified from the search, ten full-text papers met the inclusion criteria. Data extraction was completed on seven papers of high methodological quality and on three lower quality papers. Findings suggest that the key barriers which affect the development and implementation of online learning in medical education include time constraints, poor technical skills, inadequate infrastructure, absence of institutional strategies and support and negative attitudes of all involved. Solutions to these include improved educator skills, incentives and reward for the time involved with development and delivery of online content, improved institutional strategies and support and positive attitude amongst all those involved in the development and delivery of online content.

**Conclusion:**

This review has identified barriers and solutions amongst medical educators to the implementation of online learning in medical education. Results can be used to inform institutional and educator practice in the development of further online learning.

**Electronic supplementary material:**

The online version of this article (10.1186/s12909-018-1240-0) contains supplementary material, which is available to authorized users.

## Background

Medical education has many long established pedagogical approaches to learning including face to face lectures in classrooms - via a teacher-centred model [1]. This particular approach to educational practices can manifest within a teaching culture [2], becoming pervasive within an organisation or discipline, leading to a reluctance to adopt new and emerging practices and technologies. Over the last number of decades there has been a shift in medical education practice from traditional forms of teaching to other media which employ online, distance or electronic learning [3]. As described by Howlett et al. [4], “Electronic (e) or online learning can be defined as the use of electronic technology and media to deliver, support and enhance both learning and teaching and involves communication between learners and teachers utilising online content”. Online learning can provide students with “easier and more effective access to a wider variety and greater quantity of information” [5]. However, the transition from traditional to online learning is not without challenges. Increasing time constraints and demands are continually placed on students and educators alike, driving departments to find new ways of providing a more personalised, self-directed learning experience.

Medical graduates of the twenty-first century are expected to ‘hit the ground running’ [6], requiring not only a traditional clinical education but also one that is up-to-date with the latest technologies in order to ensure flexibility in a dynamic workplace. There has never been a greater need for educators, students and clinicians to continuously update their skills, to remain abreast of the changing healthcare environment and to remain ‘digitally literate’. Digital literacy has been defined as: (a) ‘The ability to use digital technology, communication tools or networks to locate, evaluate, use and create information’, (b)‘ The ability to understand and use information in multiple formats from a wide range of sources when it is presented via computers.’, (c) ‘Literacy includes the ability to read and interpret media, to reproduce data and images through digital manipulation, and to evaluate and apply new knowledge gained from digital environments’ [7]. The advent of mobile devices, Web 2.0, Web 3.0 and more recently Web 4.0 and the explosion of social media technology provides opportunities for learners to create their own personalised learning experiences. Academic faculty and tutors therefore have a crucial role in guiding and supporting the effective use of technology for such learning.

Many factors can influence whether or not an online learning programme will succeed or fail, ranging from student led factors to staff led factors [8, 9]. For example, “cultural resistances” amongst staff have previously been identified as a barrier to student engagement with technology-based education; therefore, staff focused initiatives may be key to the introduction of successful e-learning programs [8]. It has also been recognised that changes and developments in medical education are putting extra pressure on already overworked faculty [10]. When considering the implementation of e-learning within a medical school or programme robust evidence based research may strengthen one’s position when encouraging faculty to remaining abreast of technological advances. It will aid in addressing underlying concerns amongst medical faculty who may be resistant to integrating e-learning into teaching practices. In order to ensure a robust evidence base for, or against, e-learning in medical education, it is crucial that account be taken of all perspectives (student, educator, training body / school / university). To date there has been no review of the evidence on barriers and solutions from a medical educator’s perspective but there has been work completed in regards to the barriers which students face and the solutions to improve engagement with online learning [11–13]. This review therefore aims to fill this gap in the literature.

### Review aims and objectives

The overarching aim of this review was to identify and synthesize existing literature relating to the barriers and solutions to the development and implementation of online learning in medical education from a medical educator perspective. The review specifically sought:

- To evaluate existing literature relating to medical educator experience, digital literacy and/or involvement with developing and implementing e-learning tools and programmes
- To identify the barriers and solutions that restrict and aid e-learning from medical educators’ perspectives

There have been changes and new introductions into the education of medical educators regarding digital literacy and e-learning tools. These tools as shown have been developed over time and as there has been no review done so far, this is why we did the review and this paper. Also, that due to the ever changing nature of e-learning and skills needed for e-learning, staff need to develop these skills or be left behind in the digital era.

## Methods

An integrative review of the literature was conducted in order to allow for the inclusion of studies with diverse methodologies, such as those with both experimental and nonexperimental designs. The framework of Whittemore & Knafl [14] was used to enhance the rigour of the review.

### Search strategies

A search strategy was devised with input from the research team which was comprised of the six authors. The following databases were to be included: Scopus, Science Direct, Medline (Ebsco), Medline (Ovid), BioMedical Central, ERIC, Ebsco and LISA. The search engine Google Scholar was also used. Grey literature sources searched included ProQuest Dissertations & Theses (UK & Ireland), ProQuest Dissertations & Theses (A&I), University of Limerick Institutional Repository and University College Dublin Institutional Repository and a reference list was also searched for relevant studies. Boolean operators (AND, OR) were used and search terms included “online learning”, “distance learning”, “medical educators” and “digital literacy”.

The search was completed over a three month period (May to July 2016) by two researchers independently. Two searches were completed with different search strings (See Additional file 1) to ensure that all relevant papers were included. Both authors screened titles and abstracts independently into a shared online workbook. Once the initial screening took place authors compared searches to ensure that results were the same.

The identified abstracts from each search were combined resulting in an overall number of abstracts to be screened for each database, search engine and grey literature sources. The researchers used the software packages Endnote and Mendeley to organise the citations. This review was completed in a two - stage process; starting with a review of abstracts and titles. Where these met all inclusion criteria, full text articles were sourced and retrieved. All full text articles were then reviewed independently against inclusion and exclusion criteria. Consensus was reached by the research team on the final list of articles to be included.

### Inclusion and exclusion criteria

All peer-reviewed journal articles that reported empirical research, were published in English over a 10 year period from 2006 to 2016 and that focused on the medical educators’ experience of online / e-learning were included. Medical educators were defined as those teaching medical students or postgraduate trainees. Studies which specifically outlined interventions relating to improving digital literacy skills amongst medical educators were included. Studies that highlighted interventions designed to impact on engagement with online learning, the development of content and implementation in higher-level institutions were included. Qualitative, quantitative and mixed method studies were also included.

Studies that evaluated e-learning / online learning amongst populations other than medical educators were excluded. Studies that did not report empirical research or were not written in the English language were excluded.

#### Data appraisal

Data appraisal was performed by two researchers using the Critical Appraisal Skills Programme (CASP) Qualitative Research Checklist for qualitative studies [15] and National Health Medical Research Council (NHMRC) Appraisal Evidence Matrix for mixed methods and quantitative research [16]. Quantitative and mixed method studies were ranked as outlined by Andrew et al. [17], (i.e. Excellent-A; Good-B; Satisfactory-C and Poor-4). Qualitative papers were also ranked according to quality of design (Low/Medium/High) [17]. All ten papers were included in the analysis, seven of high quality and three of low quality.

#### Data extraction

Qualitative data was extracted using Supplementary Guidance Notes for Inclusion of Qualitative Research in Cochrane Systematic Reviews of Interventions [18]. Quantitative and mixed method data was extracted using an extraction tool, which included aspects of Noyes et al. [18] guidelines, informed by subject matter experts within the research team. Two researchers completed the data extraction from the eligible papers independently and reached a consensus on extracted data following this process.

#### Data analysis

Thematic analysis was the most appropriate form of data analysis for the mixed method studies included in this review. Each article was analysed and deductively coded under two headings:

1. Barriers to the development and implementation of online learning
2. Solutions to the barriers to the development and implementation of online learning.

A thematic worksheet allowed the two authors to analyse and synthesise data under qualitative and quantitative / mixed method studies independently. Thematic analysis was completed by two researchers manually and using the software package NVivo 10. Final themes were agreed by all authors.

## Results

### Search results

The initial search yielded 3101 abstracts across all sources (See Additional file 2).

**Fig. 1:**
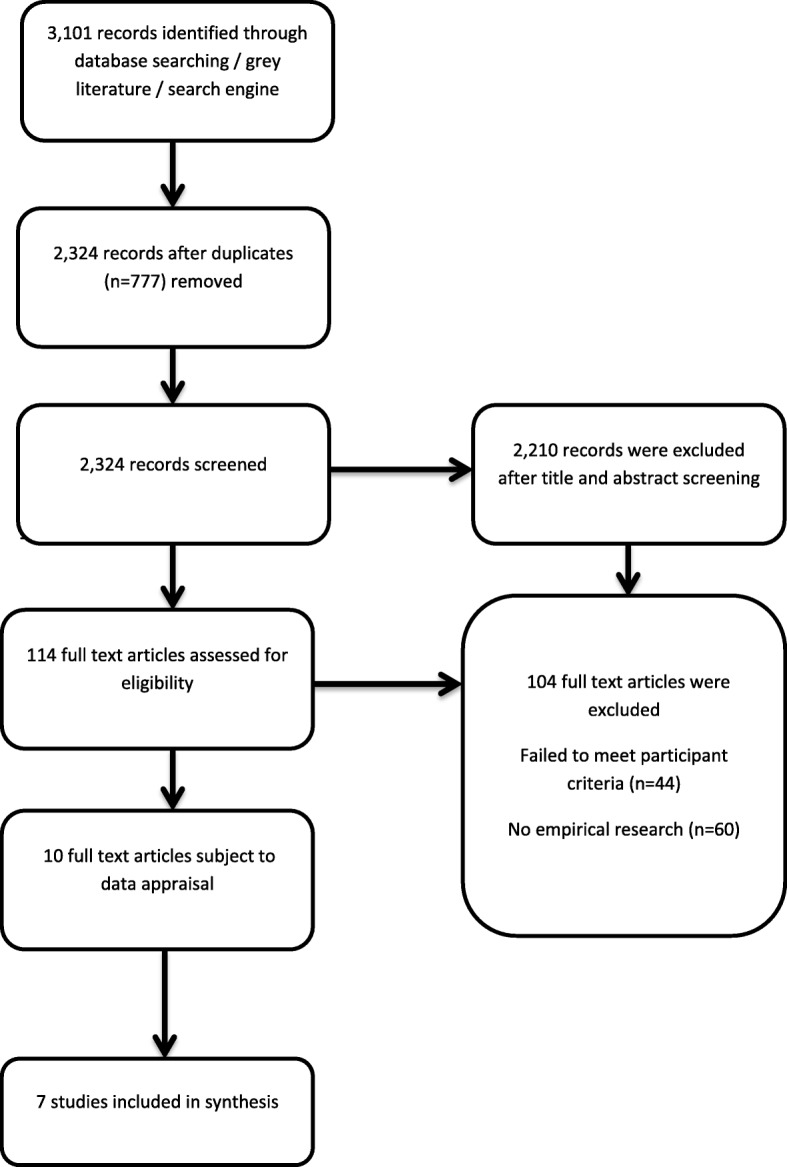
Flow diagram of study selection

A total of 2210 articles were excluded following first screening with 114 articles deemed suitable for full text review (see Fig. 1 flow diagram of study selection).

One hundred and four of these were excluded by not meeting participant criteria (*n* = 44), i.e. medical educators; or not reporting empirical research (*n* = 60). Many of the excluded studies focused on the student experience of engaging with e-learning programs / distance education and online learning. Of the 10 studies that met the inclusion criteria, these were included in final analysis. Table 1 describes the 10 studies; eight quantitative / mixed methods studies and two qualitative. Data was extracted using guidelines [15] which included aims of each study, sampling approach, participant characteristics, data collection methods and data analysis approach (see Table 1 Outline of studies included in review).

**Table 1:** Outline of studies included in review

Citation	Location	Study Design	Sampling Size	Data Collection Methods	Survey information
Bury, R.., Martin, L. & Roberts, S [27] “Achieving change through mutual development: supported online learning and the evolving roles of health and information professionals”	England, UK	Descriptive	3 participants	Literature review, interviews & tutorial programme –Triangulation	N/A
Dyrbye, L., Cumyn, A., Day, H. & Heflin, M. [20] “A Qualitative study of physicians’ experiences with online learning in a master’s degree program: Benefits, challenges, and proposed solutions”	Chicago, USA	Descriptive	71 participants – Physicians pursing a degree in higher education with online learning	Email survey with a mixed method analysis. Participation was elective.	Survey consisted of 4 structured / closed items and three open-ended items. Open-ended questions related to experiences with online courses. Closed item questions related to demographics, number of courses taken online and computing skills.
Skye, E., Wimsatt, L., Master-Hunter, T & Locke, A [28] “Developing Online Learning Modules in a Family Medicine Residency”	Michigan, USA	Descriptive	16 participants – Participants were made up of 12 faculty members and 4 residents	Web-based surveys, participant observation focus groups and pre-testing/post-testing & module evaluation	Survey consisted of 14 items with 30 evaluative statements regarding satisfaction with development process, training provided and We b authoring software. Six statements relating to author self-competence and motivation to develop modules in the future were also included. A Likert-type scale ranging from 1 = very difficult to 4 = very easy was used. Nine statements related to the level of difficulty authors faced were also included. These were scaled 1-very difficult to 1 = very easy. Questions were both open and close-ended.
Maloney, S., Haas, R., Keating, J., Molloy, E., Jolly, B., Sims, J., Morgan, P. & Haines, T (2014) “Breakeven, cost benefit, cost effectiveness, and willingness to pay for web based versus face to face education delivery for health professionals”	Victoria, Australia	Experimental	46 participants	Randomised Controlled Trial (RCT)	N/A
Brueckner, J. & Gould, D [23] “Health Science Faculty Members’ Perceptions of Curricular Integration: Insights and Obstacles”	Kentucky, USA	Descriptive	44 participants- Participants were 34 clinicians and 10 educators	Surveys at two locations	Nine item survey – gauged the perceptions of faculty regarding curricular integration in their program. Questions asked about participants’ programs current level of integration, their individual interest in increasing integration and potential solutions that could increase integration. Items were rated on a Likert scale, 1 = low interest, 5 = high interest. Questions were both open and close ended.
Niebuhr. V., Niebuhr, B., Trumble, J & Urbani M.J. [19] “Online Faculty Developments for Creating E-Learning Materials”	Texas, USA	Descriptive	27 participants- Participants were health professional educators across schools of medicine, nursing, health professionals and graduate studies	Survey & Interviews	Evaluation surveys were completed on each curriculum unit with Likert-scales, 1- strongly agreed to 5 = strongly disagree, using Survey Monkey. Questions were both open and close-ended.
Bediang, G., Stoll, B., Geissbuhler, A., Klohn, A.M., Stuckelberger, A., Nko’o, S & Chastonay, P [9] “Computer literacy and E-learning perception in Cameroon: the case of Yaounde Faculty of Medicine and Biomedical Sciences”	Cameroon, Africa	Descriptive - Cross- sectional study	1435 participants – Participants consisted of 1000 students, 275 residents and 160 lecturers	Survey	Survey was based on a validated questionnaire developed by University of Geneva. Written questionnaire was collected. Questionnaire designed to have 89 questions for lecturers and 74 for residents and students. Questions gathered information regarding access to internet, mastery of computer and medical information research strategies and knowledge and perception of e-learning.. These were both multiple choice and ranking Likert-scale style questions. These were all close-ended questions.
Attardi, S. & Rogers K (2014) “Design and implementation of an online systemic human anatomy course with laboratory”	Western University, Ontario, Canada	Evaluation	365 face to face students and 40 online students interacting with anatomy teaching assistants	Analysis of grade – incoming average grades and final anatomy grade average	N/A
Perlman, L., Christner, J., Ross, P. & Lypson, M [21] “A Successful Faculty Development Program for Implementing a Sociocultural ePortfolio Assessment Tool”	Michigan, USA	Qualitative and evaluation – More focus on the tool	Faculty mentors selected by sociocultural course director	3 Faculty workshops – focus groups	N/A
Mayer, B., Ring, C., Muche, R., Rothenbacher, D & Schmidt- Strasburger, U [35] “Creating a blended learning module in an online master study programme in oncology”	Ulm, Germany	Quantitative & Evaluation	Participants are qualified medical doctors or researchers of biomedical areas related to oncology	Evaluation of lectures – student evaluations	N/A

### Coding

**Fig. 2:**
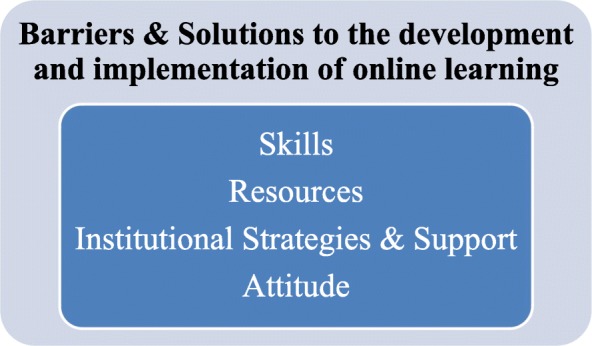
Core themes identified through the coding process

After an iterative process involving the research team, four main themes emerged. These categories are inclusive of barriers to the development and implementation of online learning and also offer solutions to those barriers (see Fig. 2 Core themes identified through the coding process).

### Core themes

#### Skills

##### Barrier – Skill deficit

Lack of skills, in particular technical skills, was found to be one of the barriers met by educators when engaging with the development and implementation of online learning [19]. Insufficient computer and typing skills [20] together with poor infrastructure can inhibit educator’s willingness or ability to engage with the development or delivery of online learning [20].

##### Solution – Engagement

In order to gain the necessary skills, it was acknowledged that engaging with e-learning, including the development of such programs, was important for gaining skills for teaching practice [19].

*“I participated so that I could learn a little bit more about teaching in an electronic environment. I think I learned quite a bit”* [19]

Perlman et al. [21] argue the importance of providing faculty with the necessary skills via training provided on the use of the ePortfolio tool. Workshops allowed faculty to learn this new skill and gain familiarity with the tool through use and from instructional design staff who were present during workshops. In order to ensure the success of the program it was crucial that faculty received the necessary training on the tool.

#### Resources

##### Barrier - time

Medical educators are already under pressure to find sufficient time to manage teaching, research and maintain a work life balance personal life commitments [22]. In this context, inadequate time to devote to the mastery, development and implementation of online learning tools can be seen as a significant barrier. This expectation of time to be invested can be seen as detrimental in an educator’s own pedagogical system in conjunction with preconceived notions that computer based tools “always take longer than expected” [19]. Interestingly, lack of time appears to be linked with lack of incentives to engage with online or e-learning [23]. Perlman et al. [21] highlight time as a barrier for faculty engaging in using an electronic ePortfolio tool. Faculty members had to invest uncompensated teaching time as they were not afforded protected administrative time due to the pilot nature of the program. It was noted that in order to ensure the effective use of such a teaching instrument, it is crucial that educators are afforded the time to become familiar with and engage with this type of tool. Faculty spent on average four to five half days of clinical work in preparing and using the tool.

##### Solution - time

The adoption of digital tools can, in fact, free up time allowing medical educators to learn concepts and to reflect on practices [20]. Furthermore, where educators are asked to spend time engaging with the development or implementation of online learning it is proposed that there be a ‘formal mechanism for faculty reward and acknowledgement for efforts…‘ [23].

##### Barrier - infrastructure

In many instances, the lack of infrastructure and technology can be seen as a barrier in medical education, typically in low-medium income countries [9]. Many of these countries lack technological basics, such as email, while others comment on the poor quality of services, such as intermittent internet access or photocopying. These technological limitations can act as a barrier to e-learning within a faculty and geographical context [9]. For example Attardi & Rogers [24] identified technical issues such as poor internet connectivity as barriers to live broadcasting of lectures in their institution in Canada. Bediang et al. [9] highlight how poor internet connectivity, Wi-Fi and access to physical infrastructure are issues which are faced in a low-income country such as Cameroon. Lakbala’s study [25] also highlights the different barriers met by health profession educators in implementing e-learning in a low-income country such as Iran. Barriers identified include limited access to computers and poor physical infrastructure.

##### Solution - cost

Maloney et al. [26] found that where a break-even analysis is completed to determine the true cost of a web-based education, the web-based approach was ‘robustly superior than a traditional face-to-face education, allowing lower number of enrolments for a program to reach its break-even point’. While this analysis might not always be an approach adopted by medical schools in developing an online programme, it is suggested as one of the ways in which one might look at the cost of establishing the correct infrastructure not as a barrier but as a potential solution to a barrier.

#### Institutional strategies & support

##### Barrier –poor communication

Where there was a lack of institutional support and limited direction as to how tools or programs would be implemented, implementation was rarely successful [27].

*“It was felt that in the early stages of the Faculty of Health, many projects were begun but the structure was missing within the Faculty to see them through*^*”*^ [27]

Implementing e-learning is often reported as a process which is adopted in polarisation; while the adoption of e-learning tools may be taking place across a number of departments in an institution, there may be a lack of interdepartmental communication which is seen as a barrier.

*“We can’t work alone! We need to work as a team”* [27]

The asynchronous environment generated is perceived as one which does not support the active exchange of ideas and shared knowledge.

*“I have found it difficult at times to have a ‘discussion’ online as you are never quite sure about the exact meaning of what people are saying.”* [20]

##### Solution - collaboration

Bediang et al. [9] found that one of the most important ways in which implementation of online / e-learning programs can be completed successfully is to include all relevant stakeholders and departments within a faculty and for new approaches to be adopted to facilitate collaboration. They specifically outlined the need for e-learning managers to put appropriate mechanisms in place in order to ‘i) have qualified and dedicated human resources, ii) allocate financial resources and iii) support of all stakeholders according to their needs’. Perlman et al. [21] noted that the provision of institutional support to faculty so that they might continue to participate in the development of online programs and for its future success is imperative.

An institutional strategy is therefore required which facilitates the implementation of key skills and the adoption of methodologies by faculty when implementing online learning [9].

#### Attitude

##### Barrier – Attitude

Negative attitude amongst educators in engaging with new technologies and tools can be seen as a barrier to the development and implementation of online learning. Educators noted feeling overwhelmed with the entire process of engaging with new tools [16] and having little patience for navigating minor technical issues [28].

*“If you ask me to peer-review something that I have no expertise in I’m reluctant to do that”* [19]

Such feelings of inadequacy, stemming from limited knowledge of, or proper training with, a particular tool may be influencing the attitude of some educators when asked to commit to implementing and developing online learning practices.

##### Solution - culture

Maintaining a positive attitude in the face of seemingly difficult to use and time consuming e-learning tools and technologies can be quite problematic. Educators involved in one study noted that it was important to try to maintain a positive attitude [20].

*“Try to maintain a positive attitude and assume that any slights or overly harsh criticism is due to the asynchronous communication and to not take itpersonally”* [20]

Adopting these new tools may in fact produce a positive experience overall and even break down preconceived notions;

*“I guess the interesting thing is that I’m old and you can teach an old dog new tricks”* [20]

Fostering a change of norms and attitudes therefore is an important solution in the development and implementation of online learning in medical education.

## Discussion

This review has thematically synthesized evidence of key barriers and solutions to the development and implementation of online learning from the medical educator’s perspective. These included skills, resources, institutional strategies and support and attitude with similar themes across many studies. This highlights the ubiquity of barriers to online learning across diverse medical education systems and speaks to a shared history of attempting to overcome them.

While positive experiences were identified, with some educators commenting on the fact that they enjoyed engaging with new tools [9, 20], there was firm emphasis on the need for strong institutional support behind such developments. Where there was a lack of institutional support and limited direction as to how such tools or programs would be implemented, implementation was rarely successful [24]. A clear institutional strategy therefore is recommended when implementing online learning [9, 26]. There is also a strong need for inter-faculty collaboration to ensure that a cohesive education is available for learners [9, 20]. Many of the themes identified in this review compliment previous studies in health profession education. For example Childs et al. [29], discussed the barriers and solutions to effective e-learning for health professionals and students and identified technical skills as both a barrier and a solution. Poor educator skills was also noted as a barrier by Pettersson & Olofsson [2]. Where issues surrounding lack of training are identified, one of the solutions proposed by Childs et al. [29] is the introduction (or improvement) of such training. Childs et al. also went so far as to recommend the enforcement of a basic computer literacy policy [29].

Pettersson & Olofsson’s study also references time as a barrier to the implementation of e-learning technologies [2]. They noted that there is limited time available for faculty to learn these new technologies which in effect damages self-confidence. The lack of time available also made faculty concerned about the pedagogical and organisational aspects of distance teaching. In order to allow educators the necessary time to learn new technologies, institutions should allow for protected time for educators to develop these skills, learn concepts and reflect on practices [20].

The increasing use of ‘inverted’ or ‘flipped classrooms’ within medical education has led to discussion on the potential of Massive Open Online Courses (MOOCs) integration into medical training. The shift from lecture based class to the use of Massive Open Online Courses (MOOCs) and the availability of open access resources pose a teaching challenge [30] with maintaining tradition often being a barrier in adopting online learning approaches by educators. Petit dit Dariel et al. [31] argues that the ‘resistance to change’ argument around e-learning technology is too simplistic. It is important to look at the intrinsic motivations that spur health professionals to either adopt or reject e-learning and how best to address these issues within specific disciplines. Specific concerns should also be recognised by institutions and departments to ensure faculty have a deeper understanding of why the change to teaching approaches is necessary and beneficial to all involved.

### Limitations of the review

There are limitations within this study which need to be acknowledged.

While four key themes, with a number of subthemes, were identified, the researchers are aware this may not be exhaustive of all the potential barriers facing medical educators engaging with online learning nor all the dominant solutions available. Despite rigorous search methodologies, it is possible that some studies were missed by the nature of the search strings used, if the keywords did not appear in the title or abstract.

Furthermore, one of the issues faced by researchers when using the database Medline (Ovid) was that of replicability. Two researchers, searching independently found conflicting results using the same search strategy and inclusion / exclusion criteria. This is a noted phenomenon as discussed by Younger & Boddy [32] where it was found that different interfaces can have an impact on the number of hits retrieved from the same database. In this instance, the team concluded that results from both searches for this database would be combined.

### Recommendations for further research

Specific themes highlighted in this review such as attitude and the importance of technical skills indicate a need for a rigorous mixed methods study focusing on the digital literacy skills of both medical educators and students. The next step in this research therefore will include a national prospective study exploring medical educators’ and medical students’ digital literacy skills within an Irish medical education setting.

### Implications for educators

Online learning has implications for educators who chose to teach via this modality including increased responsibility, the need to alter teaching style and the need to maintain meaningful ongoing communication [33, 34]. The barriers and solutions to online learning identified in this review highlight the need for significant “buy-in” from individual educators when encouraging a move to online learning. There is a need for medical educators to gain a comprehensive overview of online platforms and technologies and to understand that their own pedagogical approaches to teaching will in fact need to shift to accommodate the online environment [4].

It is also clear from the findings that institutional support when promoting this method of learning is of utmost importance and that this support should include encouraging future developments to ensure that online learning as a mode of teaching is maintained and updated to reflect the dynamic nature of information technology (IT).

## Conclusion

Online learning in medical education is a relatively new concept and one which is rapidly expanding. It is important therefore that postgraduate training bodies, medical schools and their educators are aware of the barriers and solutions to the development and implementation of type of learning and of the need for a culture to be in place which strives to promote and support the use of online learning amongst staff. In doing so, medical educators and students will be better prepared for the challenges faced in this digital age.

## Additional files

Additional file 1: Search Strategy. (PDF 14 kb)

Additional file 2: Database / Grey Literature Searches. (PDF 194 kb)

## Abbreviations

E-learning: Electronic learning
IT: Information Technology
MOOCs: Massive Open Online Courses
