# Supplementary material for: Barriers and solutions to online learning in medical education – an integrative review
Source: BMC Med Educ. 2018 Jun 7;18:130. doi: 10.1186/s12909-018-1240-0 (PMC5992716; doi:10.1186/s12909-018-1240-0)
Supplement: Supplementary file 1 — Search Strategy. (PDF 14 kb) [file 12909_2018_1240_MOESM1_ESM.pdf]

Additional file 1: Search Strategy

1. (online learning OR virtual learning OR web-based learning OR blended learning OR internet-based learning OR distance teaching) AND (medical educat* OR medical faculty) AND (development OR delivery OR implementation OR integration) AND (barriers OR obstacles) AND solutions AND digital literacy
