# Supplementary material for: Barriers and solutions to online learning in medical education – an integrative review
Source: BMC Med Educ. 2018 Jun 7;18:130. doi: 10.1186/s12909-018-1240-0 (PMC5992716; doi:10.1186/s12909-018-1240-0)
Supplement: Supplementary file 2 — Database / Grey Literature Searches. (PDF 194 kb) [file 12909_2018_1240_MOESM2_ESM.pdf]

Additional file 2: Database / Grey Literature Searches

Database / Grey Literature	Number of results
Science Direct	200
Scopus	29
BioMedical Central	23
PubMed	219
Ebsco (Academic Search Complete)	17
ERIC	11
Google Scholar	1,828
All Aboard Reference List	184
ProQuest Theses & Dissertations: UK & Ireland	57
ProQuest Theses & Dissertations: A & I	127
University of Limerick Institutional Repository	406
University College Dublin Institutional Repository	0
**Total**	**3,101**
